# A Customized Monkeypox Virus Genomic Database (MPXV DB v1.0) for Rapid Sequence Analysis and Phylogenomic Discoveries in CLC Microbial Genomics

**DOI:** 10.3390/v15010040

**Published:** 2022-12-22

**Authors:** Jane Shen-Gunther, Hong Cai, Yufeng Wang

**Affiliations:** 1 Department of Clinical Investigation, Gynecologic Oncology & Clinical Investigation, Brooke Army Medical Center, Fort Sam Houston, TX 78234, USA; 2 Department of Molecular Microbiology and Immunology, University of Texas at San Antonio, San Antonio, TX 78249, USA; 3 South Texas Center for Emerging Infectious Diseases, University of Texas at San Antonio, San Antonio, TX 78249, USA

**Keywords:** bioinformatics, disease outbreaks, monkeypox, monkeypox virus, next generation sequencing, phylogeny, poxvirus, taxonomic classification, virus database

## Abstract

Monkeypox has been a neglected, zoonotic tropical disease for over 50 years. Since the 2022 global outbreak, hundreds of human clinical samples have been subjected to next-generation sequencing (NGS) worldwide with raw data deposited in public repositories. However, sequence analysis for in-depth investigation of viral evolution remains hindered by the lack of a curated, whole genome Monkeypox virus (MPXV) database (DB) and efficient bioinformatics pipelines. To address this, we developed a customized MPXV DB for integration with “ready-to-use” workflows in the CLC Microbial Genomics Module for whole genomic and metagenomic analysis. After database construction (218 MPXV genomes), whole genome alignment, pairwise comparison, and evolutionary analysis of all genomes were analyzed to autogenerate tabular outputs and visual displays (collective runtime: 16 min). The clinical utility of the MPXV DB was demonstrated by using a Chimpanzee fecal, hybrid-capture NGS dataset (publicly available) for metagenomic, phylogenomic, and viral/host integration analysis. The clinically relevant MPXV DB embedded in CLC workflows proved to be a rapid method of sequence analysis useful for phylogenomic exploration and a wide range of applications in translational science.

## 1. Introduction

In 1958, two outbreaks of a pox-like disease in cynomolgus monkeys occurred at the Statens Serum Institut (SSI) in Copenhagen, Denmark [1]. The novel virus, which infected the captive monkeys imported from Singapore, was isolated at the SSI and so named “monkey pox virus” [1]. Over the next decade, 8 more monkeypox outbreaks befell captive monkeys imported from Southeast Asia to the Netherlands and USA [2]. In 1970, the first human case of monkeypox afflicted a 9-month-old boy from the Basankusu Territory of the Democratic Republic of the Congo (DRC) [3]. Close human contact with the large monkey population in the territory through hunting, cooking, and playing with live or dead animals were considered possible modes of transmission [3]. Between 1970 and 2017, four outbreaks of human monkeypox were reported in Africa [4]. As the 2017 Nigerian outbreak continued throughout 2018–2019, several travel-related cases from Nigeria to the United Kingdom, Israel and Singapore emerged [4]. However, human-to-human transmission did not surge until May 2022. On 23 July 2022, the World Health Organization (WHO) declared monkeypox a Public Health Emergency of International Concern (PHEIC) due to an alarming rise in cases across continents [5]. The mode of transmission had changed abruptly from animal-to-human to human-to-human (primarily sexual) contact with infectious dermal or mucosal lesions, respiratory droplets, and contaminated fomites [4]. The unprecedented outbreak reached 79,231 cases across 110 countries by 10 November 2022 [6].

The monkeypox virus (MPXV) is a large brick-shaped virus with an outer envelope and a dumbbell-shaped inner core encasing a linear, 197 kb double-stranded DNA genome [7,8,9,10]. The MPXV genome has a highly conserved central region encoding genes for viral replication, essential enzymes and structural proteins [7,8,9,10]. The terminal ends of the genome are variable and encodes genes for viral/host interactions, viral defense, and host range [7,8,9,10]. The distal ends of the genome contain inverted terminal repetitions (ITRs) covalently joined by hairpin loops [7,8,9,10]. The viral life cycle begins with virion entry into the host cell by fusion with the plasma or endocytic membrane. After uncoating, replication takes place in the cytoplasm within membraned foci called virus “factories” [7]. Approximately 10,000 genomes are synthesized per cell, but half are eventually packaged into virions [7]. Phylogenetically, two clades of MPXV, i.e., Congo Basin and West African named for its geographic origin have been recognized for notable differences in case fatality rates (>10% vs. <1%, respectively) attributable to a few disparate virulence genes [4,8,9,10].

Since the 2022 outbreak, hundreds of clinical samples have been sequenced by numerous institutions worldwide and deposited in the National Center for Biotechnology Information (NCBI) Virus repository [11]. Samples deposited in the NCBI Sequence Read Archive (SRA) repository for high-throughput sequencing (HTS) data revealed use of different sequencing platform, e.g., Illumina, Ion Torrent, and Oxford Nanopore, and viral enrichment strategies, e.g., amplicon and targeted capture [12]. Furthermore, the MPXV taxonomy of these samples were based on the International Committee on Taxonomy of Viruses (ICTV) Release 2021 with 9 ranks from “Realm” to “Species” [13]. The lack of specificity beyond “Species”, i.e., “MPXV” posed a towering barrier to lineage/sublineage differentiation between the globally deposited genomes. Adequate taxonomic depth is essential for automated metagenomic and comparative genomic analysis. To circumvent these challenges in bioinformatics (i.e., taxonomic depth and disparate HTS platforms and strategies), we developed a customized MPXV DB composed of whole genome sequences deposited in NCBI Virus [11] with a clinically relevant taxonomic structure and metadata for use in a versatile, user-friendly software. We aimed to test the utility of the database within automated workflows of the CLC Microbial Genomics Module (MGM) for MPXV whole genomic and metagenomic analysis. Ultimately, a rapid and streamlined means of sequence analysis will thrust MPXV research forward to serve a broad range of research and clinical applications.

## 2. Materials and Methods

### 2.1. Construction and Content of Customized Reference Databases

**Figure 1:**
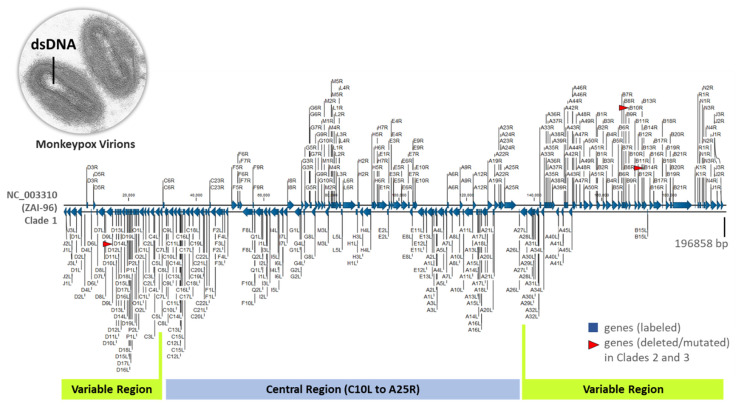
Representative Monkeypox virus genome. Linear, double-stranded, reference genome (NC_003310), ZAI-96-I-16 (MPV-ZAI) isolate from a patient during the 1996 outbreak in Zaire. The 196,858-bp sequence encodes 190 open reading frames (ORFs) with a highly conserved central region and terminal variable regions sealed in hair-pin loops on the ends. The triplet-coded, alpha-numeric nomenclature of the genes represents the Hind III restriction endonuclease fragments (alphabets), the n^th^ gene (number) counted from the left of a fragment, and the direction of transcription (R: rightward, L: leftward). Notably, three genes are deleted (D14L) or mutated (B10R and B14R) in the less virulent, prototypic Western African MPXV isolate (SL-V70) (red arrow heads) [8,9,10]. Electron micrograph of two monkeypox virions revealing dumbbell-shaped inner core [18].

MPXV RefSeq (*n* = 1) and other complete genomes (*n* = 217) from the NCBI Virus repository under Taxid: 10244 (https://www.ncbi.nlm.nih.gov/labs/virus/vssi/#/) accessed on 4 July 2022 were downloaded as GenBank files [11]. The files from human (*n* = 203) and animal (*n* = 15) hosts with a collection period from 1958 to July 2022 were imported into CLC MGM and customized for use as a database. Of note, the MPXV genomes from a Gambian pouched rat, Dormouse, and Rope squirrel collected in the USA around 2003 were identical. Only the Gambian pouched rat sequence was retained since duplication of genomes is not permissible in construction of the taxonomic profiling index in CLC. A representative MPXV genome as visualized in CLC is shown in Figure 1.

Customization involved creation of an author-defined, clinically relevant, common 7-level taxonomic nomenclature for each MPVX genome file. The current MPVX taxonomic nomenclature with 9 primary ranks by the ICTV 2021 Release is as follows: Realm: Varidnaviria; Kingdom: Bamfordvirae; Phylum: Nucleocytoviricota; Class: Pokkesviricetes; Order: Chitovirales; Family: Poxviridae; Subfamily: Chordopoxvirinae; Genus: Orthopoxvirus; Species: Monkeypox virus [13]. Due to the lack of taxonomic depth beyond species, we created a customized taxonomy based on the attributes of the Baltimore classification and recently proposed lineage/sublineage nomenclature by Happi et al. [14,15]. Specifically, we defined our 7-level taxonomic ranks as: Virus_nucleic acid type; Family; Subfamily; Genus; Species; Lineage; and Sublineage. Lineage (Clade) was numbered as “1” for Congo Basin or Central African, “2” for West African, and “3” for the 2022 Outbreak clade (a branch from the West African clade) [15]. The Sublineages (Group) for the Central African clade were roman numerals “I” through “V” and for West African clade “A” and “B” [15,16,17]. For example, the taxonomy of the MPXV RefSeq genome (Strain: Zaire-97-I-16; Accession: NC_003310) was annotated as “Virus_dsDNA_env; Chordopoxvirinae; Poxviridae; Orthopoxvirus; MPXV; 1; IV.” The customized taxonomy created in a metadata file replaced the original taxonomy of the sequence file for downstream applications as described in Section 2.2.

The Outgroup of pox genomes for comparative analysis was constructed similarly as above. Four RefSeq genomes from the NCBI Virus repository (Molluscum Contagiosum subtype 1 (NC_001731.1), Cowpox (NC_003663.2), Variola (NC_001611.1), and Vaccinia Variola) were downloaded as GenBank files and named as the “MOCOVA” reference database. The taxonomic nomenclature followed the convention as described above. Only Molluscum Contagiosum virus (MOCV) had lineages, i.e., subtype 1. All other pox viruses did not have defined lineages/sublineages so the reserved space was left empty in the 7-level taxonomic nomenclature.

### 2.2. Whole Genome Alignment Plugin, Tools, and Methods

**Figure 2:**
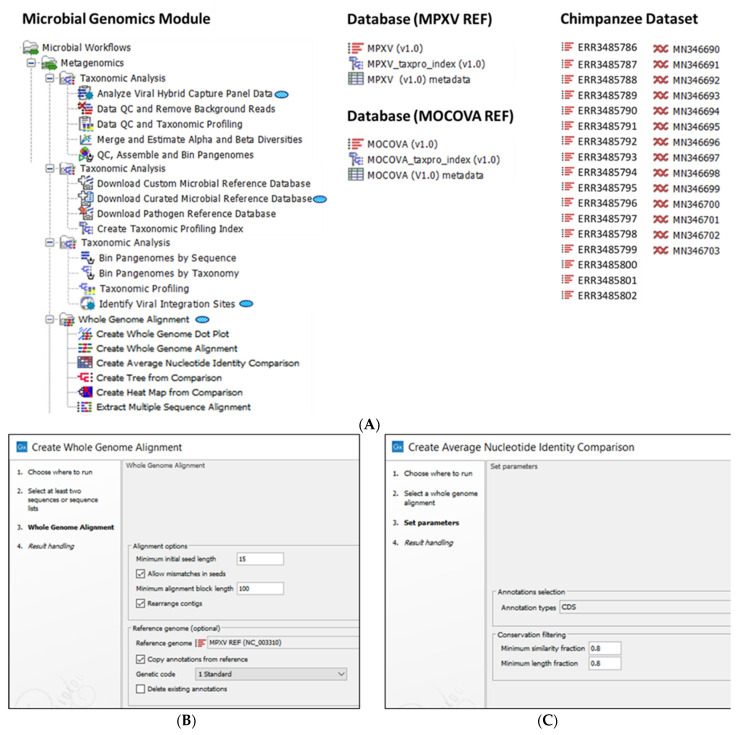
Bioinformatics methods (**A**) CLC Microbial Genomics Module, databases and dataset used for Monkeypox Virus (MPXV) Whole Genome Alignment (WGA) and comparative genomics. Primary workflows and tools used for this study are designated by the blue virus icon; (**B**) WGA workflow steps (1–4) with user-defined parameter settings for WGA (bold) and annotation, i.e., MPXV REF genome; (**C**) Create Average Nucleotide Identity Comparison workflow inputs the MPXV WGA file to quantify the similarity between genomes. The output is an autogenerated pairwise comparison matrix.

CLC Genomics Workbench 21.0.4 and CLC Microbial Genomics Module (CLC MGM) 21.0 (Redwood City, CA, USA) were installed on an HP notebook computer (specifications: Windows 10 operating system, Intel i7-7500U dual-core processor at 2.70 GHz and 8 GB RAM) for all analyses. The CLC system requirements are provided online [19]. The “Whole Genome Alignment” plugin was downloaded from within the CLC Workbench and the “Create Whole Genome Alignment” tool was used for automated data analysis (Figure 2A). The analysis consisted of 3 primary steps: (1) sequence import (*n* = 218), (2) alignment parameters selection, and (3) annotation copying from a reference genome (optional) (Figure 2B). The tool generated a WGA displaying the aligned regions between all genomes.

To add metadata to the aligned sequences after WGA, the sequences were converted into a singular sequence list using the “Create Sequence List” tool. Metadata or attributes in .xlsx format were appended to the sequence list using the “Update Sequence Attributes in Lists” tool. Our customized metadata consisted of the sequence metadata downloaded from NCBI and newly appended columns with standardized nomenclature. The metadata edited or appended included: isolate description, geolocation, continent (7-continent model), country (3-country code), host scientific name, host common name, year (sample collected), and accession number-country-year. The metadata files for MPXV and MOCOVA (Supplementary Tables S1 and S2, respectively) were also used for downstream applications, e.g., Update sequence attributes in lists and Create tree from comparison. Finally, the singular sequence list was partitioned with the “Split Sequence List” tool based on attributes, such as the sequence “Name” to revert to individual sequences.

The above sequences now annotated and enriched with metadata were aligned again with the WGA tool, however, without selection of the reference genome for an unrooted phylogenetic analysis. The WGA output was entered into the “Create Average Nucleotide Identity Comparison” tool to quantify the similarity between genomes (Figure 2A,C). For each pair of genomes, the aligned regions were identified for calculation of two measurements: (1) Alignment Percentage (AP) defined as average percentage of two genomes which is aligned, and (2) Average Nucleotide Identity (ANI) defined as average percentage of matching nucleotides for the aligned regions. The tool generated a pairwise comparison table. The pairwise comparison table (type AP or ANI) was entered into the “Create Tree from Comparison” tool for construction of a Neighbor Joining (NJ) or Unweighted Pair Group Method with Arithmetic Mean (UPGMA) tree (Figure 2A). To augment the visual information displayed by the phylogenetic tree, metadata were selected to decorate or colorize the nodes, branches, labels, and layers of metadata.

The “Create Whole Genome Dot Plot” tool (Figure 2A) was used to compare representative MPXV genomes from each of the 3 clades to get an overview of the similarities and subtle differences between them. The fundamental differences between whole genome versus classic dot plot analysis are explained in the online manual [20]. Maps of geolocations by clade were constructed using Wolfram Mathematica 13.0 (Champaign, IL, USA).

### 2.3. Utility of the MPXV Database for Sequence Analysis of Zoonotic MPXV in Fecal Samples of Chimpanzees

We used a publicly available NGS dataset of MPXV-positive, fecal samples from wild Chimpanzees of Tai National Park, Ivory Coast [21]. After DNA extraction, the DNA genomic library was enriched using a targeted-capture technique for MPXV fragments prior to paired end sequencing on the MiniSeq instrument. The zoonotic MPXV dataset of FASTQ files (*n* = 17) is available from the NCBI Sequence Read Archive (SRA) under accession numbers ERR3485786 to ERR3485802 (Figure 2A) [21]. The consensus sequences (*n* = 14) constructed from NGS reads is available from the NCBI Nucleotide database under GenBank accession numbers MN346690 to MN346702 (Figure 2A) [21]. For this study, the sequence files were directly downloaded into CLC MGM using the “Search for Reads in SRA” and “Search for Sequences at NCBI” download tools. The dataset was used to demonstrate the utility of the MPXV database for Viral Hybrid Capture Analysis (VHC) and Viral-Host Integration Site Mapping (VIS) described below.

#### 2.3.1. Viral Hybrid Capture Analysis and Workflow

The “Analyze Viral Hybrid Capture (VHC) Data” ready-to-use workflow of CLC MGM was used for automated data analysis. The analysis consisted of 4 primary steps: (1) Data import, (2) Data quality control (QC), (3) Taxonomic Profiling of reads mapping to MPXV and chimpanzee reference genomes, and (4) Low frequency variant detection (Supplementary Figure S1). Post-workflow output included tables and visualization tracks for read mapping, annotated genetic variants, annotated amino acids, and low coverage areas. The consensus sequences deposited in GenBank (MN346690 to MN346702) were used for WGA and phylogenetic inference. Of note, the consensus sequences generated by the VHC workflow may be used interchangeably.

#### 2.3.2. Viral Integration Site (VIS) Analysis and Workflow

The “Identify Viral Integration Sites (VIS)” ready-to-use workflow of CLC MGM was used for automated data analysis. The analysis consisted of 4 primary steps: (1) Data import, (2) Reads mapping to MPXV and chimpanzee reference genomes, (3) Breakpoint detection in viral and chimpanzee genomes, and (4) Gene identification surrounding breakpoint(s) (Supplementary Figure S2). Workflow outputs included tables, read mapping to host and MPXV genomes, and circular plot of viral-host genomes zoomable from the chromosome to gene level.

To prepare host genome files for the VIS workflow, the chimpanzee reference genome was accessed. The *Pan troglodytes* (chimpanzee) reference genome (Clint_PTRv2) is available from the NCBI Assembly database under the GenBank assembly accession: GCA_002880755.3 or RefSeq assembly accession: GCF_002880755.1 [22]. In the RefSeq assembly, the RefSeq accession numbers (NC_036879 to NC_036902) correspond to the 23 chromosomes [22]. For this study, the sequence files were downloaded directly into CLC MGM using the “Search for Sequences at NCBI” tool and entering the RefSeq accession numbers. The sequences were renamed “1,…, 22, and X” for each chromosome, and converted to “Chimp Genome, Gene and CDS” tracks using the “Convert to Tracks” tool for use in the VIS workflow (Supplementary Figure S2).

## 3. Results

### 3.1. MPXV DB Whole Genome Alignment (WGA) and Pairwise Comparison

**Figure 3:**
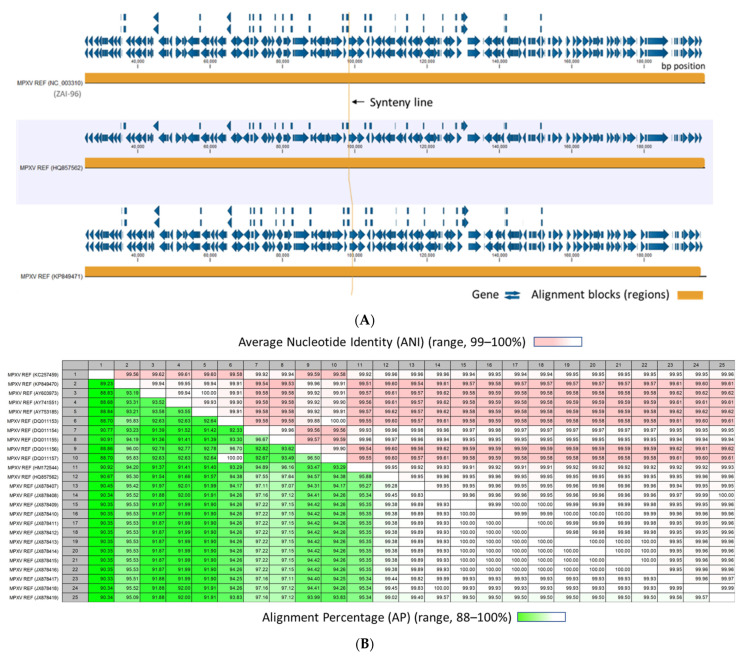
Representative views of the MPXV Whole Genome Alignment (WGA) and Pairwise Comparison Table (**A**) MPXV WGA view shows the alignments between three MPXV genomic sequences. The view of the entire alignment (218 whole MPXV genomes) is shown in Supplementary Video S1. The synteny blocks (conserved genomic regions among strains) and connected line correspond to aligned regions of the genomes (orange rectangle); (**B**) Pairwise comparison table with quantitative measures of similarity between MPXV genomes. Lower comparison matrix tabulates the Alignment Percentage (AP) or average alignment percentage between two MPXV genomes (range, 88–100%). Upper comparison matrix tabulates the Average Nucleotide Identity (ANI) or the percentage of exact nucleotide matches of the aligned regions (range, 99–100%). The lightest shades of green or pink corresponds to the highest sequence similarity (%).

After construction of the customized MPXV reference database as described in Section 2.1, WGA was performed with a runtime of 08:51 min. A representative view of the alignment between three MPXV genomic sequences is shown in Figure 3A. The synteny blocks (conserved genomic regions among strains) in the center and connected line correspond to aligned regions of the ~197,000 bp genomes (Figure 1). Zooming in on the alignments to the gene or nucleotide level allows for detailed comparison between sequences. The entire alignment (218 whole MPXV genomes) is presented as a scrolling video due to the extensive length (Supplementary Video S1).

The runtime for the “Create Average Nucleotide Identity Comparison” tool was 06:47 min. The output, i.e., pairwise comparison (PWC) table with quantitative measures of similarity between MPXV genomes is shown in Figure 3B. The lower comparison matrix tabulates the Alignment Percentage (AP) or average alignment percentage between two MPXV genomes (range, 88–100%). The upper comparison matrix tabulates the Average Nucleotide Identity (ANI) or the percentage of exact nucleotide matches of the aligned regions (range, 99–100%). In other words, the MPXV genomes have >88% similarity in the central region of the genome with nearly identical nucleotides (99–100%). The differences (<12%) are on the distal ends of the genomes.

#### 3.1.1. Phylogenetic Trees of MPXV Genomes

**Figure 4:**
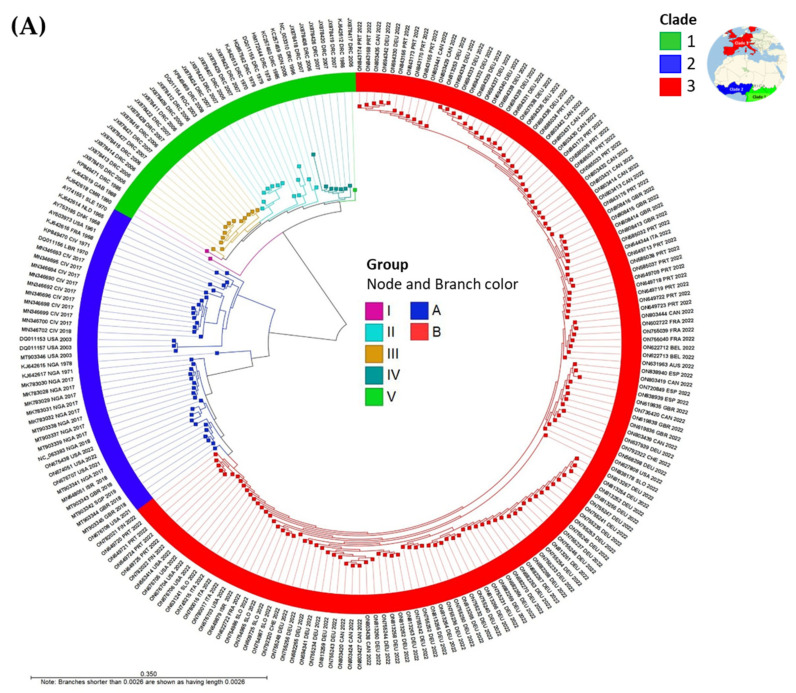

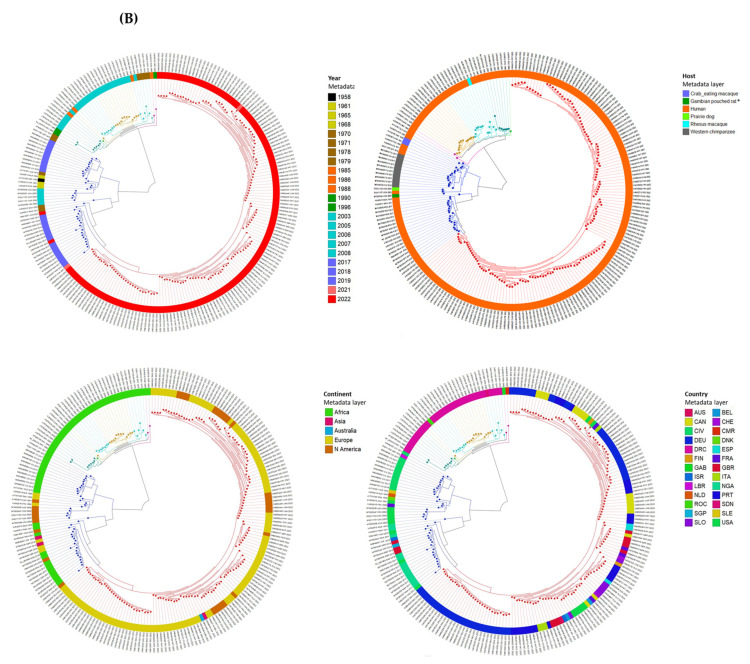
Circular phylograms of MPXV genomes by metadata for temporal, host, and spatial visualization (**A**) Phylogram of complete MPXV genomes (*n* = 218) by clades. Clades 1 and 2 originated from Central and West Africa, respectively, with divergent branches. In contrast, Clade 3 descends from Clade 2 and consists of publicly available MPXV genomes from the 2022 global outbreak. The sublineages (group) of the clades reveal the relatedness of its member samples. Three attributes of each genome (sequence accession number, 3-letter country code, and year) are displayed as the outermost ring (label) of the phylogram; (**B**) Phylograms of the MPXV genomes by metadata, i.e., year (color-coded by decades), host, continent, and country. The temporal, host, and spatial relationships reveal how the MPXV, first discovered in 1958, spilled over from mammals to humans, and spread quickly in 2022 out of Africa into Europe and North America. * The MPXV genomes of a Dormouse and Rope squirrel (USA, 2003) identical to that of a Gambian pouched rat are not shown. (The ANI NJ unrooted trees were constructed from the pairwise comparison table).

Circular phylograms of MPXV genomes were created from the PWC table using the “Create Tree from Comparison” tool in ~1 s. Metadata enriched the temporal, host, and spatial visualization of the genomes. The phylogram of complete MPXV genomes (*n* = 218) by clades is shown in Figure 4A. Clades 1 and 2 originated from Central and West Africa, respectively, with divergent branches. In contrast, Clade 3 descends from Clade 2 and consists of publicly available MPXV genomes from the 2022 global outbreak. The sublineages of Clade 1 (Groups I-V) are distinct from those of Clades 2 and 3 (Groups A and B) Figure 4A. Three attributes of each genome (sequence accession number, 3-letter country code, and year) displayed as the outermost ring of the phylogram enriched the visualization of geotemporal data.

Phylograms of the MPXV genomes by metadata, i.e., year (color-coded by decades), host, continent, and country are presented in Figure 4B. The temporal, host, and spatial relationships reveal how the MPXV, first discovered in 1958, spilled over into the human population from mammals, and spread quickly in 2022 out of Africa into Europe and North America.

#### 3.1.2. MPXV and Outgroup Genomic Comparative Analysis

**Figure 5:**
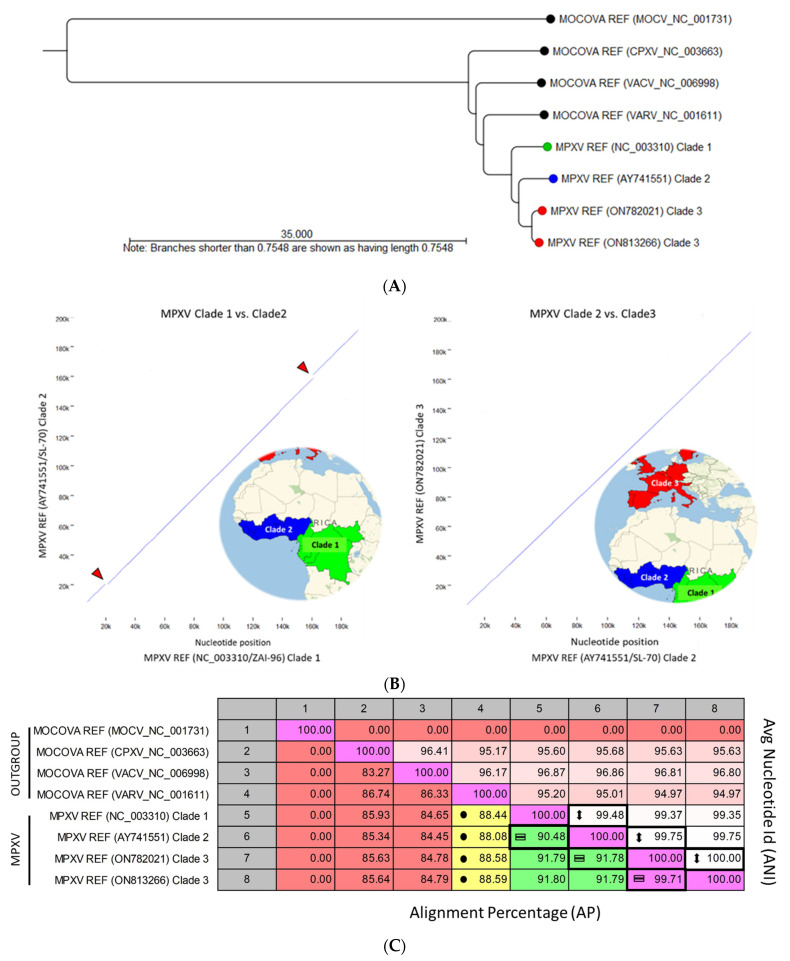
Comparative analysis of representative Monkeypox, Molluscipox, and other Orthopox viral genomes (**A**) Phylogenetic tree showing the genetic distances between MPXV and the Outgroup (MOCV, CPXV, VACV, VARV). The three clades of MPXV are distinct with intra-clade genetic evolution identified within Clade 3. See text for details regarding the representative Clade 3 samples. The NJ tree is rooted at MOCV; (**B**) Dot-plot comparison of nucleotide similarity between MPXV Clades (1 versus 2) and (2 versus 3). The unbroken diagonal represents identity between two sequences. Breaks (red arrowheads) indicate significant differences. Specifically, MPXV REF (ZAI-96) Clade 1 possesses 3 genes which are deleted (D14L) or mutated (B10R and B14R) in Clade 2 located at the breaks near positions 20k and 168k, respectively. Clades 2 and 3 are genetically similar without notable breaks; (**C**) Pairwise comparisons show 90% to 92% alignment similarity (=) between MPXV Clades, and > 99% nucleotide identity (↕) within aligned regions. In contrast, the MPXV and outgroup genomes differed by >12% (<88.6% AP) with smallpox (VARV) highlighted (●). AP, alignment percentage; ANI, average nucleotide identity; MOCV, Molluscum contagiosum virus; CPXV, Cowpox virus; VARV, Variola virus; VACV, Vaccinia virus.

Comparative analysis of representative Monkeypox and Outgroup (MOCV, CPXV, VACV, VARV) viral genomes was performed after WGA with MOCV as the designated reference genome (runtime: 03:53 min.). The pairwise comparison and tree construction took <5 s (runtimes: 3 and 1 s., respectively). The API NJ phylogenetic tree rooted at MOCV shows the genetic distances between MPXV and the Outgroup members (Figure 5A). The three clades of MPXV are distinct, and intra-clade genetic evolution is identified within clade 3 (red nodes) with divergent branches. The two representative samples selected from clade 3 were the most distant genomes from the current dataset.

The runtime for “Create Whole Genome Dot Plot” was ~ 1 s for each pair of genomes. Dot-plot comparison of nucleotide similarity between MPXV Clades (1 versus 2) and (2 versus 3) is shown in Figure 5B. Breaks in the diagnonal line (red arrowheads) indicate significant differences. Specifically, MPXV REF (ZAI-96) Clade 1 possesses 3 genes which are deleted (D14L) or mutated (B10R and B14R) in Clade 2 located at the breaks near positions 20k and 168k, respectively. Clades 2 and 3 are genetically similar without discernable breaks. Genome pairwise comparisons show 90% to 92% alignment similarity (=) between MPXV Clades, and >99% nucleotide identity (↕) within aligned regions (Figure 5C). In contrast, the MPXV and outgroup genomes were dissimilar by >12% (<88% AP) with smallpox (VARV) being the most similar (~88%).

#### 3.1.3. Utility of MPXV DB for Sequence and Phylogenetic Analysis using a Hybrid-Capture NGS Dataset Derived from Chimpanzee Fecal Samples

**Figure 6:**
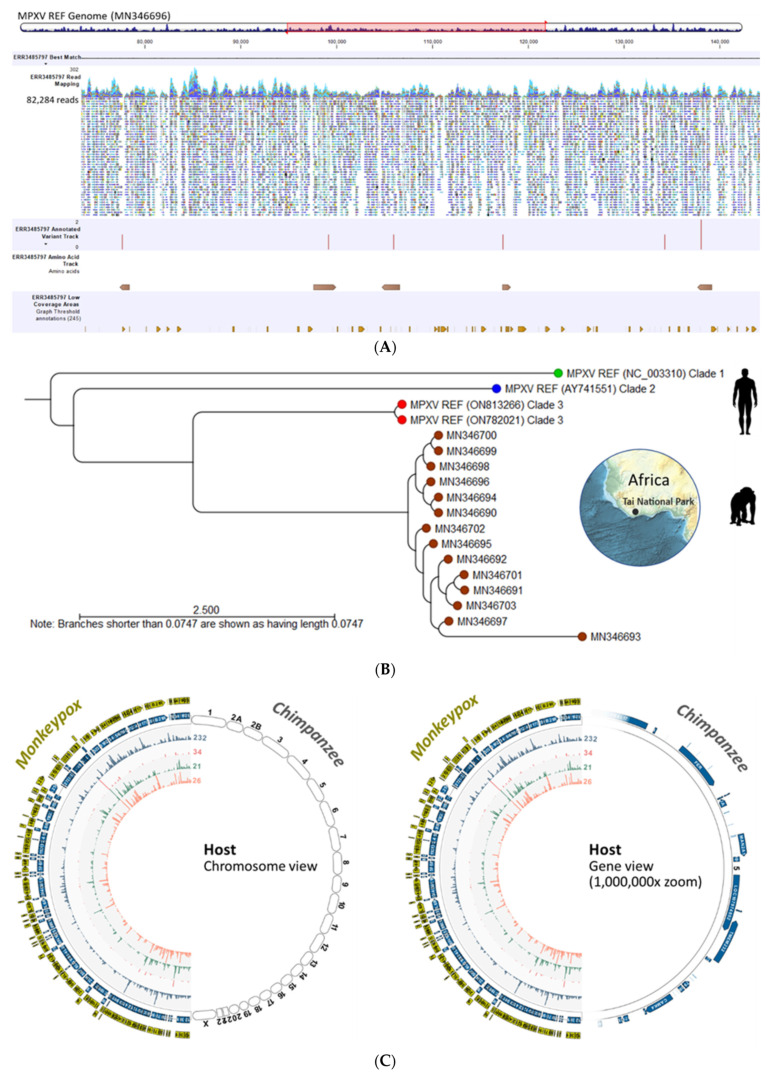
Utility of the MPXV database for sequence and phylogenetic analysis using a Chimpanzee dataset. (**A**) A deep-sequenced MPXV sample (ERR3485797) extracted from Chimpanzee feces is analyzed with the Viral Hybrid Capture (VHC) automated workflow. The resultant VHC track list displays (top to bottom): read mapping against the closest MPVX reference genome, annotated variant track, MPXV amino acid track, and low coverage areas; (**B**) Phylogenetic analysis of the consensus sequences derived from the Chimpanzee dataset (*n* = 14) reveals significant divergence from the human MPXV. Among the Chimpanzee MPXV sequences, two clades are revealed; (**C**) Viral-host Integration Site (VIS) analysis for sample (ERR3485797) visualized as circular plots. The bipartite MPXV/Chimpanzee genomes in chromosome view (left) and gene view (1,000,000 × zoom, right) did not reveal any sites of viral integration. (Virus–host integration linkages are designated by bi-directional curvilinear lines between the virus and host genomes).

A representative, deep-sequenced MPXV sample (ERR3485797) extracted from Chimpanzee feces was analyzed with the Viral Hybrid Capture (VHC) automated workflow (runtime: 10:36 min). The resultant VHC track list displays (top to bottom): read mapping against the closest MPVX reference genome, annotated variant track, MPXV amino acid track, and low coverage areas (Figure 6A). The track list shows 82,284 paired-reads mapped onto the linearized reference genome with auto-detected low coverage areas for rapid quality assessment. The entire length of the mapped reads is shown in Supplementary Video S1. The consensus sequence generated from the reads mapping may be utilized for WGA and phylogenetic analysis. Finally, variants are easily localized within the track, and variant(s) information, e.g., type of genetic alteration, amino acid change, and non- or synonymous mutation are summarized in a table.

After WGA, phylogenetic analysis of the consensus sequences derived from the Chimpanzee dataset (*n* = 14) revealed significant divergence from the human MPXV (Figure 6B). Among the Chimpanzee MPXV sequences, two clades were revealed. More importantly, the MPXV genomes from the same locale, Tai National Park, were genetically distinct with divergent branches in the phylogenetic tree. This may be explained by intra-host mutagenesis and viral microevolution by APOBEC3 as proposed by Isidro et al. [23].

The “Identify Viral Integration Sites (VIS)” ready-to-use workflow of CLC MGM was used to map potential viral-host integration sites. Using the representative sample (ERR3485797), the VIS analysis generated a circular plot as shown in Figure 6C. The bipartite MPXV/Chimpanzee genomes in chromosome view (left) and gene view (1,000,000 × zoom, right) did not reveal any sites of viral integration. Virus–host integration linkages are manifested as chimeric reads. If present, the chimeric reads are designated by bi-directional curvilinear lines between the virus and host genomes within the circular plot [24]. The dynamic functions of the VIS circular plot are presented in Supplementary Video S1.

## 4. Discussion

In this study, we developed a customized, whole genome MPXV DB for use within the CLC MGM. To examine the relationships among the NCBI-derived MPXV genomes (*n* = 218) of the database, the CLC WGA Plugin with complete set of alignment and analytical tools were used. We were able to visualize and examine the whole genome alignment, pair-wise comparison table, and phylograms within 16 min, collectively. The WGA informed genetic differences between the MPXV clades which confers host specificity and virulence [10]. The pair-wise comparison table generated in ~7 min revealed the percentage of inter- and intra-clade nucleotide differences critical for studying viral evolution. The expanded, customized metadata of the MPXV DB enhanced visualization of the temporal, host, and spatial relationships of the phylogenetic trees. In essence, the trees revealed how the MPXV, first discovered in 1958, spilled over from mammals to humans, spread out of Africa in 2022, and dispersed rapidly across continents.

We also developed the MOCOVA DB for comparative genomic analysis. The WGA, pairwise comparison, and phylogenetic tree between MOCOVA and representative MPXV genomes determined the genetic distances between pox genomes in under ~5 min. More importantly, we were able to investigate the genomic similarly between MPXV from the 2022 outbreak and smallpox (VARV RefSeq) (88%), as well as the more virulent MPXV Clade 1 (92%). Furthermore, the whole genome dot plot identified significant genetic differences between MPXV clade 1 and 2 near positions 20k and 168k with genes believed to confer virulence [7,8,9,10]. Lastly, the MOCOVA DB may also be used to identify other viral causes of vesicular lesions by sequencing (Sanger or NGS) to avoid delayed diagnosis and inappropriate empirical therapy [25]. Taken together, the efficient, stepwise methodology described herein is both relevant and valuable to the study of emerging viral infectious diseases, outbreak investigations, and global surveillance.

The utility of the MPXV DB was also demonstrated by using a Chimpanzee excrement NGS dataset [20]. By incorporating curated MPXV and chimpanzee genome databases within CLC MGM workflows, we were able to process hybrid-capture NGS data simply by inputting the FASTQ files, selecting the reference genomes, and setting the parameters. The VHC mapping and track list with zoomable visualization provided effortless inspection of mapped regions, variants, and low coverage areas at the nucleotide and amino acid levels. The MPXV consensus sequences generated from the VHC workflow may be aligned for studying the MPXV sub-lineages and evolutionary relationship between samples. Finally, VIS workflow was performed simply by inputting the FASTQ files and selecting the viral and host reference genomes. The VIS dynamic, dual genome, circular plot facilitated visualization and identification of potential virus/host integration sites. Since the MPXV replicates exclusively in the cytoplasm, the absence of viral/host integration events in this dataset was expected. Finally, the utility of excrement for MPXV detection has been extrapolated and demonstrated recently in human wastewater-based surveillance (WBS) from Northern California and a few other states [26].

The strength of the methods developed herein is the integration of a customized MPXV database with automated workflows for viral whole genome and metagenomic analysis. We strived to provide the user with a rapid, streamlined tool to investigate newly sequenced or publicly deposited samples to advance scientific discovery, as well as, for surveillance and monitoring. Monkeypox has been considered a neglected tropical disease for decades [4]. This is reflected in the scanty bioinformatics literature dedicated to MPXV. Of the 3 published articles found in PubMed spanning from 2004 to 2019, all emerged from the Viral Bioinformatics Research Centre, Victoria, British Columbia [27,28,29]. In their 2019 publication, Tu and Upton used several lab-developed bioinformatics tools (e.g., Viral Genome Organizer (VGO), Genome Annotation Transfer Utility (GATU), JDotter, and Base-by-Base (BBB)) to annotate, dot plot and compare whole MPXV genomes. The enormous efforts dedicated to developing the software programs for the bench virologist are commendable and appreciated by the scientific community. Nevertheless, for the non-bioinformatist, i.e., virologists, epidemiologists, and physician-scientists, the time and effort required to learn and use these sophisticated programs are overwhelming and cost inefficient. Automated bioinformatics tools and pipelines are desperately needed to reduce the barrier to studying rapidly evolving, emerging infectious diseases.

We acknowledge that our study has limitations, that is HTS reads generated by other sequencing platforms, e.g., Ion Torrent and Oxford Nanopore Technologies (ONT) were not included in this study for comparison. To bridge this gap, we intend to expand our testing to other sequencing platforms. Additionally, new viral enrichment hybrid capture kits (e.g., QIAseq xHYB MPXV Panel) require further bioinformatics end-user testing [30]. Although we did not identify viral/host genomic integration in the Chimpanzee dataset, investigating such events using hybrid capture NGS is important. It is currently unknown if viral/host integration occurs in HIV-infected and/or immunocompromised individuals.

## 5. Conclusions

In conclusion, the MPXV and ancillary MOCOVA databases integrated with CLC automated workflows provide a rapid, streamlined means of sequence analysis. The pipelines for viral metagenomic and whole genomic analysis will facilitate discoveries and advancements in monkeypox research.

## Data Availability

The Chimpanzee MPXV NGS dataset is publicly available from NCBI SRA (https://www.ncbi.nlm.nih.gov/sra?term=ERP116902, accessed on 10 November 2022) under study accession number ERP116902. The consensus sequences of the same dataset are available from NCBI Virus (https://www.ncbi.nlm.nih.gov/labs/virus/vssi/#/, accessed on 10 November 2022) under GenBank accession numbers MN346690 to MN346702.

## Supplementary Materials

The following are available online at https://www.mdpi.com/article/10.3390/v15010040/s1, Figure S1: Viral hybrid capture (VHC) workflow, Figure S2: Viral Integration Site (VIS) workflow, Table S1: MPXV v1.0 metadata, Table S2: MOCOVA v1.0 metadata, Video S1: Dynamic visualization of the MPXV whole genome alignment, VHC and VIS outputs.

## Abbreviations

AP: alignment percentage; ANI, average nucleotide identity; CLC MGM, CLC Microbial Genomics Module; COPV, Cowpox virus; dsDNA, double-stranded DNA; DB, database; env, enveloped; HTS, high-throughput sequencing; ICTV, International Committee on Taxonomy of Viruses; MOCV, Molluscum Contagiosum virus; MOCVA, Molluscum Contagiosum, Cowpox, Variola, Vaccinia; MPXV, Monkeypox virus; NCBI, National Center for Biotechnology Information; NGS, Next-generation sequencing; NJ, Neighbor Joining; ONT, Oxford Nanopore Technologies; PHEIC, Public Health Emergency of International Concern; REF, reference genome; SRA, Sequence Read Archive; VACV, Vaccinia virus; VARV, Variola virus; VHC, Viral Hybrid Capture; VIS, Viral Integration Site; WGA, whole genome alignment; WHO, World Health Organization.
