# Supplementary material for: A Customized Monkeypox Virus Genomic Database (MPXV DB v1.0) for Rapid Sequence Analysis and Phylogenomic Discoveries in CLC Microbial Genomics
Source: Viruses. 2022 Dec 22;15(1):40. doi: 10.3390/v15010040 (PMC9861985; doi:10.3390/v15010040)
Supplement: Supplementary file 1 [file viruses-15-00040-s001.zip › FIG S1 VHC settings.pdf]

A

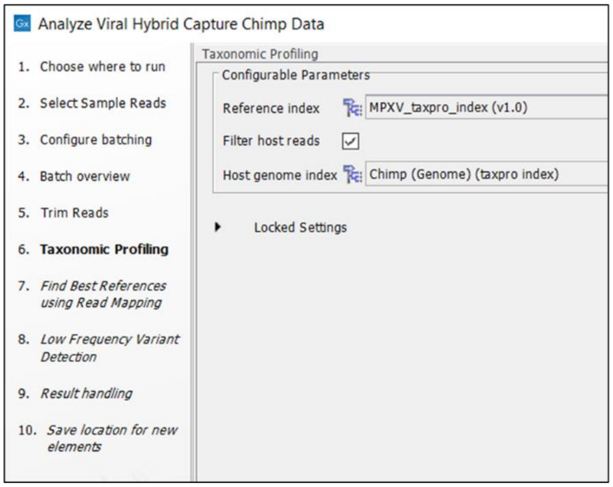
Analyze Viral Hybrid Capture Chimp Data
1. Choose where to run
2. Select Sample Reads
3. Configure batching
4. Batch overview
5. Trim Reads
6. Taxonomic Profiling
7. Find Best References using Read Mapping
8. Low Frequency Variant Detection
9. Result handling
10. Save location for new elements
Taxonomic Profiling
Configurable Parameters
Reference index
MPXV_taxpro_index (v1.0)
Filter host reads
Host genome index
Chimp (Genome) (taxpro index)
Locked Settings

B

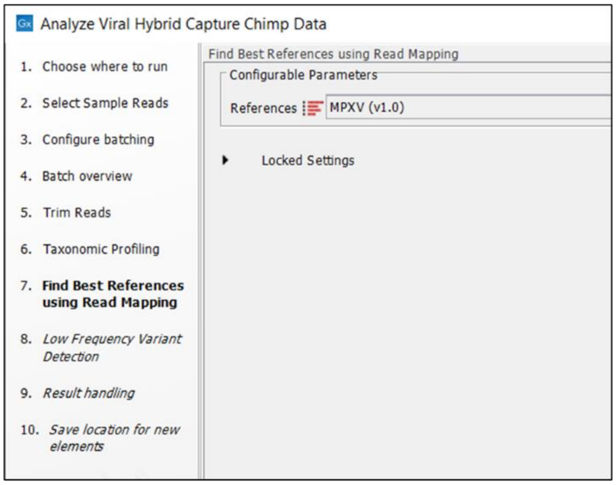
Analyze Viral Hybrid Capture Chimp Data
1. Choose where to run
2. Select Sample Reads
3. Configure batching
4. Batch overview
5. Trim Reads
6. Taxonomic Profiling
7. Find Best References using Read Mapping
8. Low Frequency Variant Detection
9. Result handling
10. Save location for new elements
Find Best References using Read Mapping
Configurable Parameters
References
MPXV (v1.0)
Locked Settings

C

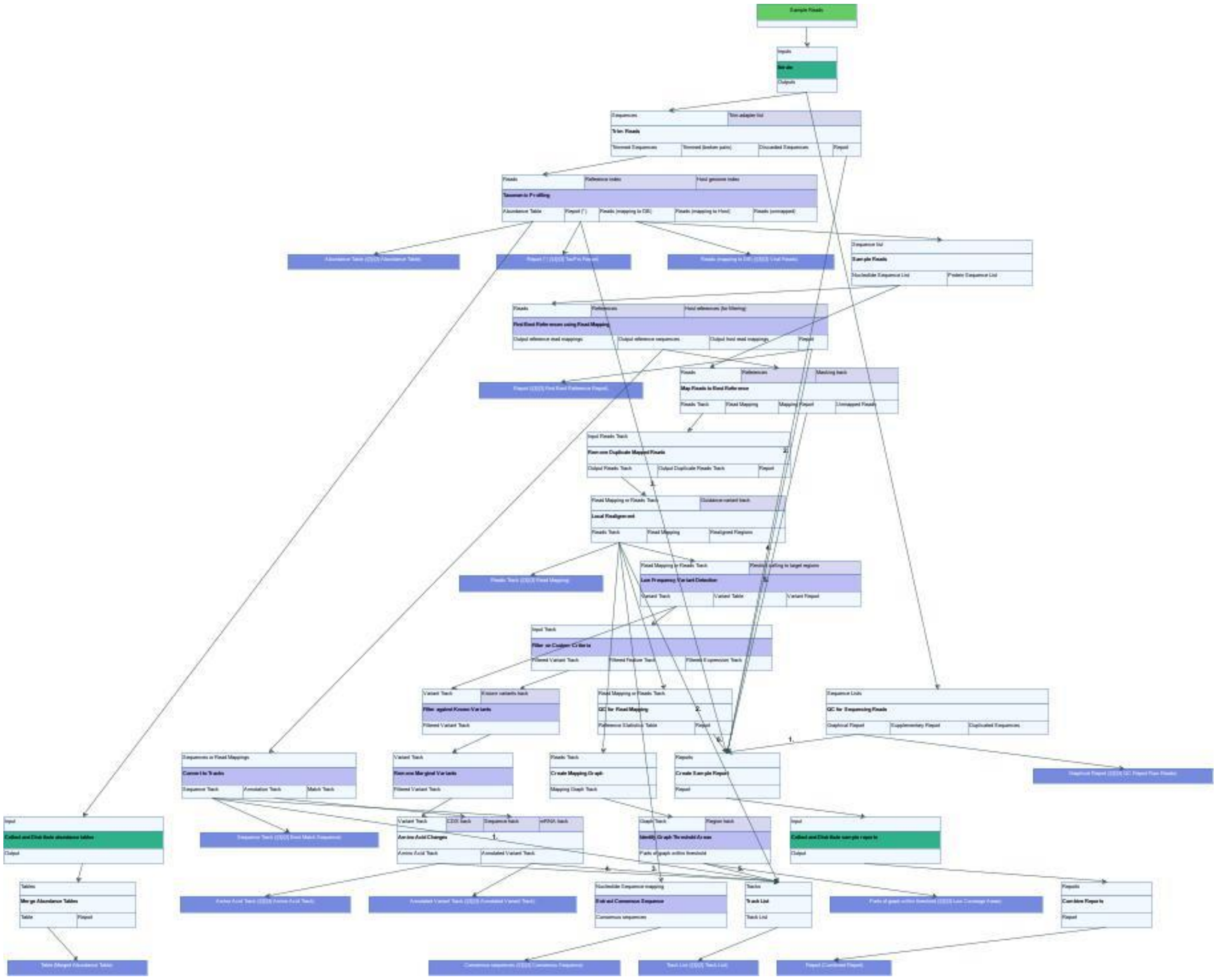

Supplementary Figure S1. Viral Hybrid Capture (VHC) analysis. (**A**) VHC workflow steps (1-10) with user defined reference indices for Taxonomic Profiling (bold), i.e., Reference index (MPXV v1.0) and Host genome index (Chimpanzee), (**B**) MPXV (v1.0) database as a sequence list is selected for Reads Mapping, (**C**) Detailed view of the entire VHC workflow used for automated analysis of the Chimpanzee hybrid capture NGS dataset [21].
