# Supplementary material for: A Customized Monkeypox Virus Genomic Database (MPXV DB v1.0) for Rapid Sequence Analysis and Phylogenomic Discoveries in CLC Microbial Genomics
Source: Viruses. 2022 Dec 22;15(1):40. doi: 10.3390/v15010040 (PMC9861985; doi:10.3390/v15010040)
Supplement: Supplementary file 1 [file viruses-15-00040-s001.zip › FIG S2 VIS settings.pdf]

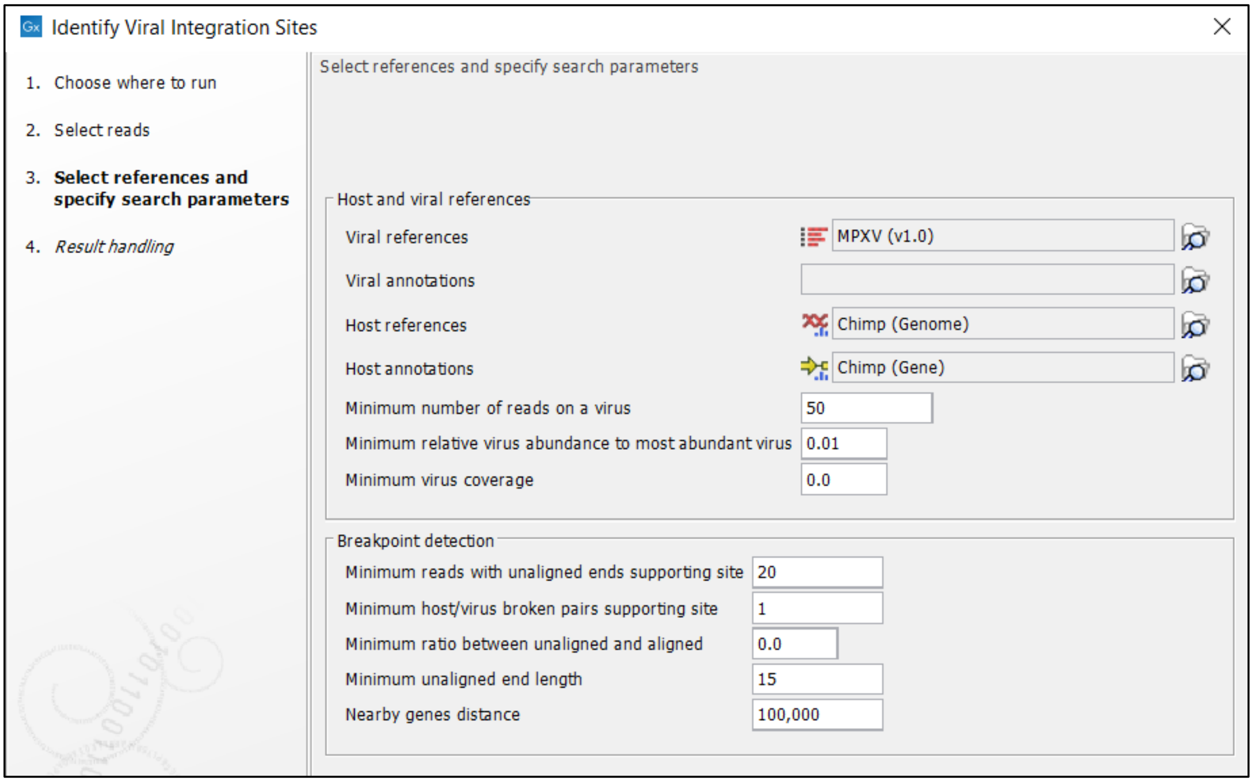

Supplementary Figure S2. Viral Integration Site (VIS) analysis. VIS workflow steps (1-4) with user defined references for the host (Chimpanzee genome and gene) and the virus (MPXV v1.0 genome database). The numerical parameters were set to default as shown. The settings as displayed were used to analyze the Chimpanzee dataset [21].
